# Editorial: Plasmid transfer-mechanisms, ecology, evolution and applications

**DOI:** 10.3389/fmicb.2022.993628

**Published:** 2022-08-16

**Authors:** Chin-Yi Chen, Clay Fuqua, Charlene R. Jackson, Kristina Kadlec, Eva M. Top

**Affiliations:** ^1^United States Department of Agriculture, Agricultural Research Service, Wyndmoor, PA, United States; ^2^Department of Biology, Indiana University, Bloomington, IN, United States; ^3^United States Department of Agriculture, Agricultural Research Service, Athens, GA, United States; ^4^Dairy Herd Consulting and Research Company (MBFG), Wunstorf, Germany; ^5^Department of Biological Sciences, Institute for Bioinformatics and Evolutionary Studies, University of Idaho, Moscow, ID, United States

**Keywords:** plasmid, conjugation, mobilization, horizontal gene transfer, Type IV secretion system (T4SS), plasmid transfer, antibiotic resistance

Plasmids are important carriers of genes involved in virulence, resistance to antibiotics and heavy metals, and metabolism of compounds, among other specialized functions. The process whereby plasmids spread among bacteria through cell-cell contact is called conjugation, or conjugative transfer. In order to limit the spread of antibiotic resistance to pathogens or improve the spread or persistence of beneficial plasmids in biotechnological or agricultural applications, we need to better understand the factors that determine plasmid spread and persistence in diverse environments. This Research Topic collected a wide range of papers concerning plasmid transfer, including the mechanisms and regulation of conjugative transfer, plasmid maintenance and interactions within a host or in the environment, as well as characterization of plasmids carrying virulence and resistance determinants that could potentially impact public health or agriculture.

## Plasmid transfer mechanisms and regulation

Several studies published in this Research Topic focused on various aspects of conjugative transfer mechanisms. While conjugation is well understood for a few model plasmids, there are still multiple outstanding questions related to its regulation, efficiency, effects of co-residing plasmids, and the host range of conjugative plasmid transfer. Expression of the conjugative transfer genes is often tightly regulated by plasmid-encoded, as well as chromosomal, transcriptional regulators but detailed kinetics of gene expression in donor vs. recipient cells is not clear. Zygotic induction is the transient activation of plasmid-encoded genes that occurs during the early stages of conjugation. Miyakoshi et al. provided new insights into the early, differential gene expression of RP4, a highly efficient conjugative IncP-1 plasmid in *Pseudomonas putida*. Following the approach of Althorpe et al. (1999), they analyzed the transcriptomes of a combination of isogenic Rif^R^ and Rif^S^ mating strains in the presence of RNA polymerase inhibitor rifampicin. Operons in the leading region of the transfer strand that are involved in stable plasmid maintenance (e.g. *kfr, kor, kle, kla*) were strongly induced in the recipient (transconjugant) cells within 10 min of a conjugation event. Conversely, on the 3' end of the transfer strand, the *traJIH* operon which encodes the relaxase and other auxiliary proteins of the RP4 plasmid, was upregulated in the donor cells. Since the *traJ* promoter is the earliest to be transferred, the relaxase may be expressed in the donor cell after regeneration of the *oriT*-flanking region and likely to displace the autogenous repressors around *oriT*.

Regulation of plasmid conjugative transfer is an active area of investigation. For broad host range IncP plasmids, the *korA* and *korB* regulators are well known to impact rates of conjugative transfer (Zatyka et al., 1998). In this Research Topic, the tryptophan biosynthesis intermediate indole, an established, self-produced regulatory signal in *Escherichia coli*, was reported to inhibit IncP conjugative transfer by upregulating the *korA-korB* genes (Xiong et al.). The antibiotic ciprofloxacin is known to repress *korA* and *korB* expression, and stimulate plasmid transfer, so perhaps indole could be used to mitigate this effect and decrease horizontal transfer.

Population density also regulates and affects conjugative transfer. For plasmids of the *Rhizobiaceae* such as species of *Rhizobium* and *Agrobacterium*, plasmid conjugative transfer is often regulated by self-produced quorum sensing signals known as acyl-homoserine lactones (AHLs) (Gordon and Christie, 2014). Barton et al. described here how two co-resident plasmids in *A. tumefaciens* with fully functional AHL quorum sensing systems cross-regulated their conjugative transfer in a very complex control network. In the mammalian pathogen *Enterococcus faecalis*, conjugative plasmids and other mobile elements are well known to transfer antibiotic resistance and virulence functions. For these systems, conjugative transfer is controlled in part by recipient-produced peptide pheromones that activate conjugative transfer functions in donors through clumping of the non-motile *E. faecalis* cells (Haemig and Dunny, 2008). In a highly promiscuous class of these pheromone-regulated plasmids, resistance to oxazolidinone-type antibiotics was demonstrated to be effectively transferred among *E. faecalis* clinical isolates, thereby imparting resistance to the clinically used linezolid antibiotic on these highly transmissible plasmids (Zou J. et al.).

Another important factor in the fate of a plasmid is its ability to maintain itself in a bacterial population in the absence of selection for the plasmid. For example, the persistence of multi-drug-resistance plasmids in the absence of antibiotics varies greatly between species or even strains of the same species (De Gelder et al., 2007; Kottara et al., 2018). The interest in intracellular effects of co-residing plasmids on plasmid evolution, spread and persistence is rapidly growing. Using a mathematical model, Gama et al. tested how the interaction between plasmids, both intracellularly and intercellularly, impacted the persistence of a focal plasmid: whether through effects on host fitness, conjugative transfer rate, or rate of loss during cell division. They concluded that there was a hierarchy amongst these interaction parameters. Plasmid interactions affecting host fitness favored plasmid persistence more than those affecting conjugative transfer rate and lastly plasmid loss during cell division. These results together with previous studies (Gama et al., 2017a,b) suggest that interactions between different plasmids can favor their persistence in bacterial communities. Hülter et al. also showed that the success of a plasmid can be determined by intracellular competition between plasmids at the level of segregation during cell division. That success may even depend on the order in which the plasmids arrive in the host.

An important mechanism by which plasmids can affect each other's transfer success is plasmid exclusion, by which recipients prevent conjugative delivery from a donor (Garcillán-Barcia and de la Cruz, 2008). Oluwadare et al. studied a common subtype of IncF-type plasmids in *Salmonella* isolates carrying the *spvB* gene, which encodes an ADP-ribosylating toxin involved in virulence. The authors demonstrated a common plasmid exclusion activity in isolates harboring this SpvB-encoding plasmid for the conjugative delivery of other IncF plasmids. The exact mechanism remains unclear, but the study implicated a role for the plasmid-borne *traS* gene, found to impart plasmid exclusion in other systems.

Another growing field is the study of regulatory interactions within a cell between plasmid and chromosome and vice versa. For example, the transfer of F-like plasmids is activated by the plasmid-encoded TraJ and chromosomally encoded ArcA proteins *via* the promoter P_Y_, whereas it is silenced by the nucleoid structuring protein H-NS. To better understand the role of these proteins in P_Y_ promoter activation, Bischof et al. performed *in vitro* DNA binding studies using these purified proteins to identify more details of this complex regulatory mechanism. In another study, Zoolkefli et al. screened the *E. coli* knock-out collection (the Keio collection) for mutants in donor chromosomal genes that increased conjugative transfer of IncP plasmids. Three mutated genes, a regulator (*fmrR*) and two genes likely to be involved in iron-sulfur cluster metabolism (*sufA* and *iscA*), were identified, leading to the proposal of a regulatory model by which these genes converge their regulatory impact on IncP conjugation genes.

In the last two decades, the number of studies that showed how the cost of a plasmid to its bacterial host can be rapidly ameliorated through either chromosomal or plasmid mutations exponentially increased (for a recent overview, see Brockhurst and Harrison, 2021). Here Kawano et al. showed that mutation in a gene encoding a stress-inducible small RNA (sRNA) or its promoter, drastically reduced the fitness cost of harboring the broad-host-range resistance plasmid RP4. Strikingly, the chromosomal *parI* gene, known to be upregulated under stress response, was also upregulated upon entry of the plasmid. They then further showed that mutations that downregulate or disable the production of this anti-sense RNA lowered the fitness cost to the host.

Some small plasmids, although not capable of self-transmission, can be mobilized by other conjugative plasmids. These mobilizable plasmids carry minimally an *oriT*, and some also encode a mobilization relaxase and accessory proteins that can interact with the relaxase and/or Type IV Secretion System (T4SS) proteins of other conjugative plasmids to facilitate transfer. Garcillán-Barcia et al. focused on the relaxase MOBQ4 of an understudied group of mobilizable plasmids in enterobacterial clinical isolates. They identified the helper conjugative plasmids responsible for the dissemination of these plasmids and showed that co-resident plasmids from different MOBQ4 subgroups did not negatively or positively affect their respective abilities to be mobilized.

Moreover, as shown by one study here, conjugation can be used as a gene delivery tool in various biotechnology applications. Samperio et al. showed that broad-host-range plasmids can be used to deliver DNA from *E. coli* to Gram-positive bacteria. Examples were lactobacilli for which transformation with DNA had not been successful so far.

Mathematical models can be very helpful to explain experimental observations and begin to predict the fate of plasmids in microbial communities. For those interested in an overview and some history of mathematical modeling approaches that have been developed over the years to explain and predict plasmid dynamics, we recommend reading Hernández-Beltrán et al.

## Plasmids in plant-associated bacteria and natural communities

This Research Topic includes multiple papers on the impact and dissemination of plasmids in bacteria associated with plants. Phytopathogenic bacteria are well known to often carry their virulence functions on conjugal plasmids. In the plant-associated Gram-positive bacterial genus of *Rhodococcus*, virulent isolates cause leafy gall or witches' broom diseases on a number of different plants. Virulence was demonstrated to be plasmid encoded, but there has been some controversy about which plasmid genes are involved in disease incidence and by which mechanisms (Savory et al., 2017). Savory et al. in this issue demonstrated that a plasmid-borne AraC-type regulator called FasR is required for virulence, but not the plasmid genes it is known to regulate that drive cytokinin biosynthesis. Comparison of gene content from the plasmids of multiple virulent *Rhodococcus* isolates suggests that in addition to *fasR*, several other virulence functions known as *att* genes are conserved, but not the FasR target genes. This led to a virulence model in which FasR regulates chromosomal genes, that along with the *att* genes, impart virulence on plants. As the *Rhodococcus* plasmids are conjugative, these functions can be transferred to other rhodococci to distribute virulence functions in the environment. For the well-studied plasmid-based virulence of *Agrobacterium tumefaciens* in crown gall and related plant diseases, the genes required for virulence are well established and carried on the tumor-inducing (Ti) plasmid. The Ti plasmid is conjugative between agrobacteria, and this is regulated by the plant interaction and a quorum sensing system encoded by the Ti plasmid. In this issue, Barton et al. revealed that in some *A. tumefaciens* strains, the co-resident megaplasmid (called At) is also conjugative and encodes its own quorum sensing system. The At plasmid exerts a complex synergistic regulatory impact on Ti plasmid conjugation *via* regulation and shared conjugation gene functions.

Nitrogen fixation genes of the legume-symbionts rhizobia are also plasmid-encoded and these plasmids are conjugatively proficient (Lagares et al., 2015). Rhizobia re-engineer plants to form nitrogen fixation structures called nodules where they perform their symbiotic activity. In a remarkable study, Bañuelos-Vazsquez et al. showed that the so-called Sym plasmid of *Rhizobium etli* was transferred to diverse bacterial community members in the developing nodule and that some of these transconjugants consequently gained the ability to drive nitrogen fixation. Some transconjugants gained the intact replicating plasmid, whereas others apparently integrated the Sym plasmid genes into the resident genome. This study suggested significant plasmid-driven complexity for nitrogen fixation capacity in legume-associated communities.

Plant rhizospheres are conducive environments for horizontal transmission of plasmids. Three different papers in this Research Topic examined the prevalence and transfer of conjugative plasmids in the rhizosphere. Hall et al. used a *Pseudomonas fluorescens* strain as the source of and as a conduit for plasmids that confer resistance to mercury in soil. They found abundant levels of plasmid transfer and observed the survival of *P. fluorescens* only in the presence of mercury due to its ability to acquire mercury resistance from the community. In a different study, mercury resistance genes (as well as catabolic gene loci) were also found to be commonly associated with broad host range conjugative IncP plasmids in microbial communities of lettuce (Shintani et al.). These plasmids did not carry the antibiotic resistance genes often found on IncP plasmids in communities of wastewater and sludge, where human and animal waste is processed. In contrast, IncP plasmids were not isolated from communities associated with tomato plants even with high mercury levels suggesting that the plant host may play a role in plasmid ecology in these environments. Finally, a previously cryptic class of IncP-type conjugative plasmids called PromA plasmids (for promiscuous A) were captured from Gram-negative soil bacteria (*Hydrogenophaga*) found in different agricultural soils in Belgium (Werner et al.). Multiple PromA plasmids have been shown to lack accessory genes, and thus their functional consequence beyond driving conjugative transfer of other plasmids was unclear (Van der Auwera et al., 2009). Several isolated plasmids here were found to confer degradation of the plant phenylurea herbicides, such as linuron, suggesting that PromA plasmids may be driving exchange of the catabolic genes in soil communities.

Another feature of plasmids that may influence their prevalence in natural communities, such as those associated with plants, is their ability to modulate biofilm formation of their host bacteria (Ghigo, 2001). Gama et al. evaluated 11 naturally occurring conjugative plasmids from *E. coli* and determined their impact on biofilm formation. They found that a good portion of the isolated plasmids enhanced biofilm formation, and that this required active conjugation, but others may have inhibitory effects. Although it is difficult to correlate biofilm enhancement with a specific plasmid-encoded function, it is clear that many plasmids can have this effect, and thus it may have an impact on their costs and benefits in natural populations.

## Plasmids carrying antibiotic resistance determinants

Dissemination of antimicrobial resistance genes among both pathogenic and commensal bacteria is often mediated by mobile genetic elements including plasmids. Knowledge of plasmids harboring resistance genes and the extent of transfer in bacterial populations is essential to development of alternatives to combat antimicrobial resistance. This is especially important as new resistance mechanisms to clinically important antimicrobials are emerging in bacteria previously not known to carry those resistance genes. An example of this is highlighted in a review article of transfer mechanisms and associated antimicrobial resistance in *Salmonella* by McMillan et al. Strains of *Salmonella* Infantis from poultry carrying a mosaic multidrug resistance (MDR) pESI-like plasmid (plasmid for Emerging *Salmonella* Infantis) have recently emerged causing infections in humans (Aviv et al., 2014; Tate et al., 2017; Brown et al., 2018). The plasmid contains *bla*_CTX−M−65_, which encodes an extended-spectrum β-lactamase (ESBL), conferring resistance to third generation cephalosporins used to treat salmonellosis in humans. Yang et al. described another type of mosaic plasmid from *Salmonella* Derby formed by excision of a chromosomally located MDR fragment flanked by IS elements. The excised MDR fragment was then combined with a conjugal plasmid *via* homologous recombination.

Several other papers also focused on the analysis of plasmids of different Inc groups responsible for resistance to beta-lactams. Other than the predominant IncF plasmids harboring a *bla*_NDM_ gene, IncX, IncA/C, IncH and two plasmids of unknown Inc types were described among clinical carbapenem resistant *E. coli* isolates from China by Zou H. et al.. Fu et al. had a closer look on plasmids of different Inc types carrying a *bla*_CTX−M_ gene originating from *Salmonella* Enteritidis from Shanghai: *bla*_CTX−M−55_ gene was most commonly found (in 32 of 38 cases) and in several cases flanked by the insertion sequence IS*Ecp1* and ORF477. Among the rare *E. coli* isolates with resistance to ampicillin or cephalosporins from wildlife in Guadeloupe (resistant *E. coli* isolates were identified in 48 of 884 animals), an IncI1 plasmid with *bla*_CTX−*M*−1_ belonging to the sequence type ST3 was described in detail (Guyomard-Rabenirina et al.). Zhang et al. described a plasmid carrying a resistance gene to the novel phosphonic drug class fosfomycin. Liu et al. reported a conjugative tetracycline resistance plasmid pTS14 that proved to be very stable in the host. The study dealing with colistin resistance plasmids focused on the identity and transferability of plasmids carrying the colistin resistance gene *mcr-1* (Majewski et al.). Also included is an article on the analysis of plasmids in Gram-positive bacteria, including virulence and resistance gene-carrying plasmids, in 222 *Staphylococcus aureus* isolates from retail meat (Neyaz et al.).

Two papers in this Research Topic focused on basic characterization of plasmids conferring antimicrobial resistance in the gut microbiota. Both the opportunistic pathogen *Raoultella ornithinolytica* from healthy adults (Wang S. et al.) and *Klebsiella pneumoniae* from a Red Kangaroo (Wang X. et al.) carried MDR IncF type plasmids with antibiotic resistance phenotypes transferable to an *E. coli* recipient in mating experiments. Another study analyzed 114 completely sequenced *E. coli* isolates from pigs and farm workers in Vietnam, transferable plasmids carrying ESBL genes and other resistance genes were identified, whereas no direct transmission of the resistance genes between animals and humans was observed (Hounmanou et al.). Law et al. showed that wastewater biosolids served as a reservoir of Prom A and IncP-1 antibiotic resistance plasmids that can transfer their resistance genes to pathogens and commensals, and that some of those plasmids can persist in these novel hosts without antibiotic selection. These studies underscore the importance of monitoring antibiotic resistance plasmids in asymptomatic animals and people, as well as in the environment.

A great deal of fundamental research has led to multiple approaches that are aimed at inhibiting plasmid transfer or accelerating plasmid loss from populations of pathogens with antibiotic resistance plasmids. Vrancianu et al. wrote a comprehensive review on these various chemical and biological approaches that can be used to eliminate mobile genetic elements as carriers of antibiotic resistance genes.

## Concluding remarks

This Research Topic highlights the active and dynamic area of research on plasmid transfer. Plasmids are important in many different contexts and in distinct environments. Their prevalence and transmissibility make them consistent contributors to specialized bacterial phenotypes such as virulence, antibiotic resistance, exotic metabolism and symbiotic functions. However, plasmids are clearly selfish genetic elements that are under natural selection to maintain and propagate themselves in bacterial populations. Their genetic content, however, can have tremendous impacts on the bacterial hosts in which they reside, including the ability of these bacteria to drive beneficial and detrimental interactions with animals, plants, and other host organisms. Further improving our understanding of plasmids and their transfer mechanisms will continue to inform new strategies of bacterial control and offer new avenues for their biotechnological application.

## Author contributions

All authors listed have made a substantial, direct, and intellectual contribution to the work and approved it for publication.

## Funding

C-YC and CJ were supported by the U.S. Department of Agriculture, Agricultural Research Service, Food Safety In-House Projects. CF was supported by NIH grant RO1 GM120337 and the Indiana University Faculty Research Support Program (FRSP). ET was partially supported by the National Institute of Allergy and Infectious Diseases at the National Institute of Health (grant number R01 AI084918).

## Conflict of interest

Author KK was employed by MBFG. The remaining authors declare that the research was conducted in the absence of any commercial or financial relationships that could be construed as a potential conflict of interest.

## Publisher's note

All claims expressed in this article are solely those of the authors and do not necessarily represent those of their affiliated organizations, or those of the publisher, the editors and the reviewers. Any product that may be evaluated in this article, or claim that may be made by its manufacturer, is not guaranteed or endorsed by the publisher.

## Author disclaimer

The opinions in this Editorial are those of the author(s) and should not be construed to represent any official USDA or U.S. Government determination or policy. USDA is an equal opportunity provider and employer.
